# Seed-competent tau monomer initiates pathology in a tauopathy mouse model

**DOI:** 10.1016/j.jbc.2022.102163

**Published:** 2022-06-22

**Authors:** Hilda Mirbaha, Dailu Chen, Vishruth Mullapudi, Sandi Jo Terpack, Charles L. White, Lukasz A. Joachimiak, Marc I. Diamond

**Affiliations:** 1 Center for Alzheimer’s and Neurodegenerative Diseases, Peter O'Donnell Jr. Brain Institute, University of Texas Southwestern Medical Center, Dallas, Texas, USA; 2 Department of Pathology, University of Texas Southwestern Medical Center, Dallas, Texas, USA

**Keywords:** tauopathy, seed-competent monomer, pathology, prion, tauopathy mouse model, size-exclusion chromatography, ACN, acetonitrile, AD, Alzheimer’s disease, MS, mass spectrometry, PTM, posttranslational modification, SEC, size exclusion chromatography, TBS, Tris-buffered saline

## Abstract

Tau aggregation into ordered assemblies causes neurodegenerative tauopathies. We previously reported that tau monomer exists in either inert (M_i_) or seed-competent (M_s_) conformational ensembles and that M_s_ encodes strains, that is, unique, self-replicating, biologically active assemblies. It is unknown if disease begins with M_s_ formation followed by fibril assembly or if M_s_ derives from fibrils and is therefore an epiphenomenon. Here, we studied a tauopathy mouse model (PS19) that expresses full-length mutant human (1N4R) tau (P301S). Insoluble tau seeding activity appeared at 2 months of age and insoluble tau protein assemblies by immunoblot at 3 months. Tau monomer from mice aged 1 to 6 weeks, purified using size-exclusion chromatography, contained soluble seeding activity at 4 weeks, before insoluble material or larger assemblies were observed, with assemblies ranging from n = 1 to 3 tau units. By 5 to 6 weeks, large soluble assemblies had formed. This indicated that the first detectable pathological forms of tau were in fact M_s_. We next examined posttranslational modifications of tau monomer from 1 to 6 weeks. We detected no phosphorylation unique to M_s_ in PS19 or human Alzheimer’s disease brains. We conclude that tauopathy begins with formation of the M_s_ monomer, whose activity is phosphorylation independent. M_s_ then self assembles to form oligomers before it forms insoluble fibrils. The conversion of tau monomer from M_i_ to M_s_ thus constitutes the first detectable step in the initiation of tauopathy in this mouse model, with obvious implications for the origins of tauopathy in humans.

Tauopathies such as Alzheimer’s disease (AD), chronic traumatic encephalopathy, frontotemporal dementias, and related disorders are each characterized by ordered assemblies of the microtubule-associated protein tau (1). We have proposed that transcellular propagation mediated by tau prions underlies their relentless progression (2, 3, 4, 5, 6, 7), as have others (8, 9). According to this model, aggregates that form in one cell escape to gain entry to connected or adjacent cells, where they act as templates for their own replication. Tau normally binds microtubules (10, 11) and does not form detectable aggregates. The initial cause of tau assembly formation in tauopathies is unknown, and it could be that high concentrations of monomer coalesce to form “seeds,” *i.e.*, forms that serve as templates for ordered assembly growth. Indeed, some have proposed that tau undergoes liquid–liquid phase separation (12, 13, 14, 15), which could theoretically initiate assembly formation through molecular crowding. By contrast, our prior work has suggested that a seed-competent tau monomer (M_s_) exists in a conformational ensemble that is distinct from inert tau monomer (M_i_) (16, 17). Another study is consistent with this idea (18), although this concept is not widely accepted. We have recently developed methods to create M_s_ from M_i_ using recombinant protein (16, 19, 20), and we have also isolated it from human tauopathies (16, 17). Intriguingly, M_s_ adopts multiple seed-competent conformational states that serve as templates to create defined tau prion “strains” (17), which are tau species that faithfully replicate their structure *in vivo*, and produce defined patterns of neuropathology (5, 6, 21). We have proposed that a critical conformational change from M_i_ to M_s_ represents the first step in the aggregation process (16), that M_s_ encodes strains (17), and we have also reported methods to produce M_s_ *in vitro* (20). However, it is unknown whether M_s_ precedes or follows the formation of large assemblies *in vivo*—a critical question for the origin of tauopathies.

We previously developed methods to purify full-length tau monomer from brain tissue using a combination of immunopurification and size exclusion chromatography (SEC) (16). This readily discriminates tau monomer from larger assemblies. We have now used this approach to determine when M_s_ first appears in a transgenic model of tauopathy and what is its relationship to higher-order assemblies. We also compare tau monomer from AD *versus* control patients and test whether phosphorylation correlates with M_s_ seeding activity.

## Results

### Insoluble tau and seeding activity *versus* neurofibrillary pathology

**Figure 1:**
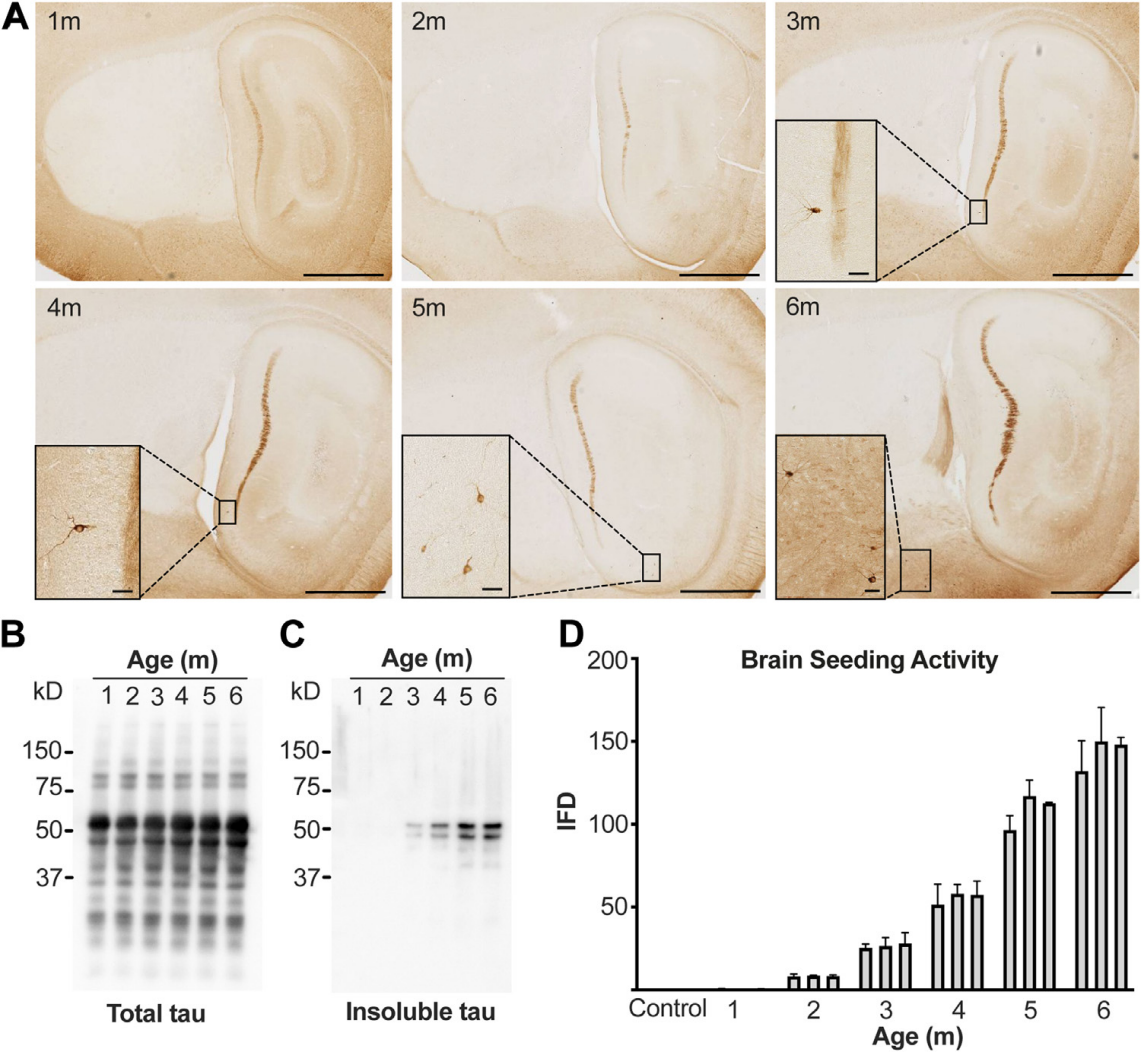
**Neurofibrillary pathology and seeding activity of insoluble tau in aging PS19 mice.***A*, sagittally sectioned hemibrain of PS19 mice aged 1 to 6 months immunostained by AT8 antibody, which highlights rare neurofibrillary tangles in 3 months in lateral sections of hippocampus and increases by age. Representative images are shown. The main window scale bar represents 1 mm, the zoomed-in window scale bar represents 30 μm. In medial sections of the hippocampus, neurofibrillary tangles were only observed in 6 months (not shown). *B*, Western blot of PS19 contralateral hemibrain homogenate of aging mice shows no difference in total tau expression level at all ages from 1 to 6 months. Tau runs slightly larger than 50 kD. Nonspecific bands, including the high-molecular-weight bands, are observed when total brain lysate is run on SDS-PAGE gel. *C*, SDS-PAGE of detergent-insoluble tau extracted from PS19 mice brain (three for each age mixed and loaded on the gel) shows insoluble tau is detectable at 3 months and increases with age. Polyclonal rabbit anti-human tau antibody was used to blot for tau in *B* and *C*. *D*, seeding activity (reported as integrated FRET density, IFD) of detergent-insoluble tau appears in mice at 2 months and increases with age. Each *bar* represents one mouse, with technical triplicates.

PS19 mice express full-length (1N4R) tau containing a P301S missense mutation that causes dominantly inherited tauopathy in humans (22). They have been extensively characterized by our laboratory and others and develop tau neurofibrillary pathology at approximately 6 months of age. We repeated this analysis, staining sections at 1 to 6 months with AT8 (23), an anti-phospho-tau antibody (pS202/pT205), using sagittally sectioned hemibrains (Fig. 1*A*) and saving the other halves for biochemistry (below). In lateral sections neurofibrillary tangles were very rare at 3 months, slightly increased at 4 months, and easily detectable at 6 months. We consistently observed neurofibrillary tangles only at 6 months (Fig. 1*A*), consistent with our prior work (24). Using the contralateral hemibrain from each animal, we analyzed brain homogenates for total tau by Western blot (Fig. 1*B*) and observed no change between 1 and 6 months. Detergent (sarkosyl) extraction of tau is commonly used to detect fibrillar aggregates, so we compared sarkosyl-insoluble fractions at different ages. We observed insoluble tau by Western blot at 3 months (Fig. 1*C*), several months before the appearance of obvious cellular tau pathology.

To determine when detergent-insoluble seeding activity appears, we tested each of the fractions in a well-established cellular biosensor assay based on expression of tau repeat domain containing a single disease-associated mutation (P301S) fused to cyan and yellow fluorescent proteins (24). We observed no seeding at 1 month and detected minimal activity at 2 months that grew steadily thereafter (Fig. 1*D*).

These experiments established that in PS19 mice overall human tau levels were constant between 1 and 6 months, insoluble seeding activity occured at 2 months, insoluble tau was apparent at 3 months by Western blot, and abundant neurofibrillary pathology did not appear until 5 to 6 months.

### Soluble tau assemblies appear at 4 to 6 weeks

**Figure 2:**
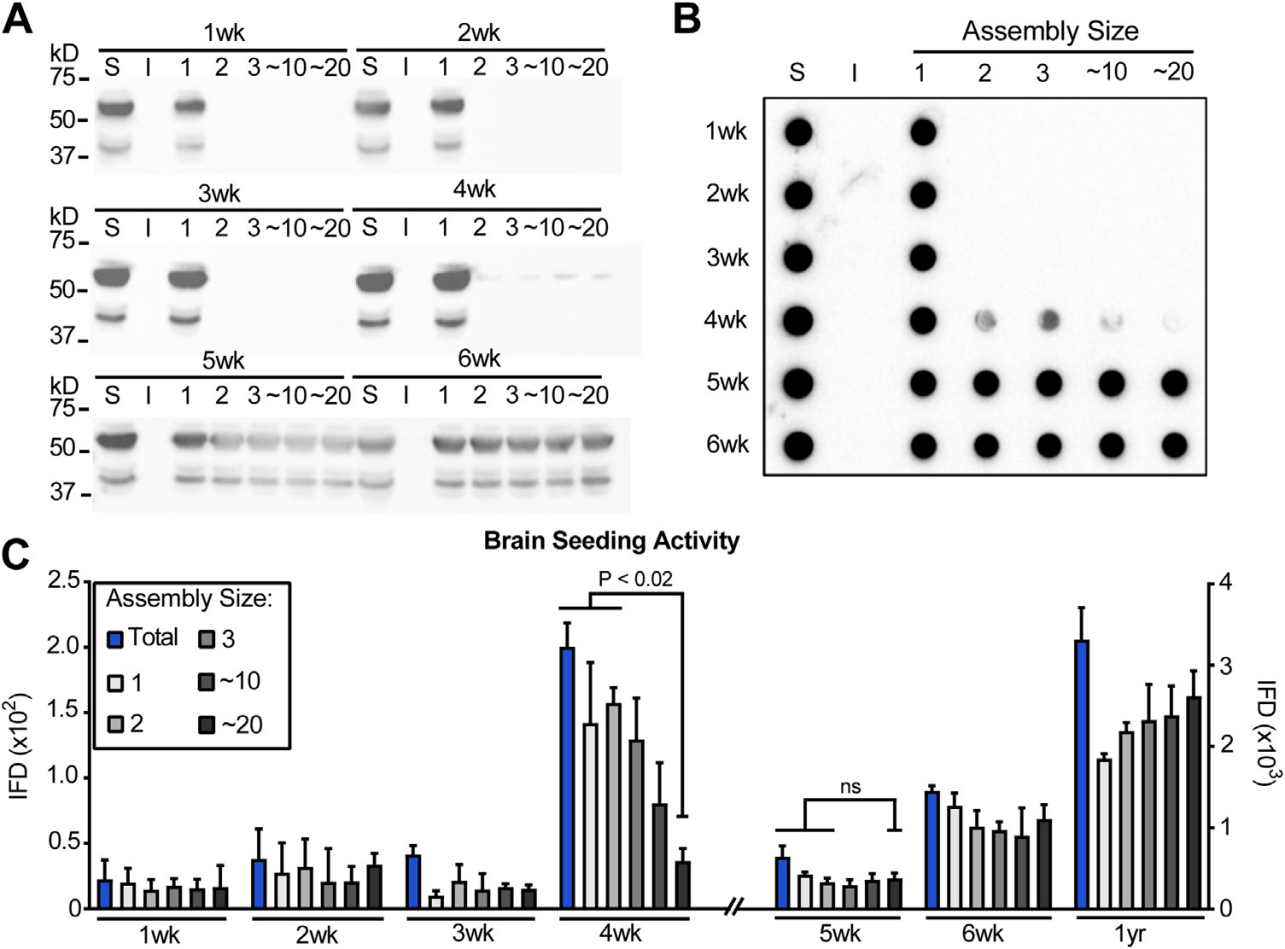
**Seeding activity of soluble tau in PS19 mice brain at 1 to 6 weeks.** To determine the assembly state of tau before formation of insoluble tau, we created PS19 brain homogenate from mice aged 1 to 6 weeks, clarified them by centrifugation at 21,000*g*, and resolved them by size exclusion chromatography. *A*, SDS-PAGE was used to analyze S = total soluble tau, I = sarkosyl-insoluble tau, or assemblies of various sizes ranging from n = 1 to ∼20-mer. *B*, similar analyses were carried out using dot blot. Polyclonal rabbit anti-human tau antibody was used to blot for tau in *A* and *B*. Note that at 4 weeks small amounts of tau assemblies became apparent, whereas the dominant form was monomer. *C*, the 21,000*g* supernatant (T), and tau assemblies were seeded into RD-CFP/YFP biosensors. Seeds consistent with tau monomer appeared at 4 weeks, with dimer and trimer assemblies, whereas 10-mer and 20-mer were much less abundant (*p* < 0.02 for 20-mer). By 5 and 6 weeks seeds of all sizes were highly abundant. Assembly size: 1 = monomer, 2 = dimer, 3 = trimer, ∼10 = ∼10-mer, ∼20 = ∼20-mer. Error bars = S.D. Statistics: two-tailed *t* test. Note different IFD scales for weeks 1 to 4, weeks 5, 6, and 1 year. Image of full Western blot is in Fig. S2. IFD, integrated FRET density.

Having established the onset of large, insoluble tau assemblies, we extended our study to younger mice ranging from 1 to 6 weeks (n = 3 per age) to characterize precisely the small changes in assembly state from monomer to dimer, trimer, ∼10-mer, and ∼20-mer. We have previously determined that M_s_ produced *in vitro* exists in dynamic equilibrium with dimers, and potentially trimers (20). We combined the three brains at each time point and extracted sarkosyl-soluble and -insoluble fractions that we analyzed by Western blot. We detected no change in soluble or insoluble tau in this time frame (Fig. 2*A*). We then resolved the supernatant from a 21,000*g* centrifugation using SEC with a Superdex 200 column, according to our established methods (16, 25). The assembly state of tau in each fraction was estimated by running gel filtration standards (Bio-Rad) on the same SEC column, in methods we have previously validated (Fig. S1) (25). Our prior work determined that there is no detectable cross-contamination between fractions that contain multimers and those that contain tau monomer (25). We performed Western blots for purified tau using polyclonal anti-tau antibody (Agilent). We observed no larger tau assemblies until 4 weeks of age, and these increased in abundance between 4 and 5 weeks (Fig. 2*A*). We also used 2-μm filter dot blot to characterize the fractions, which confirmed the findings regarding soluble *versus* insoluble tau and the prevalence of small assemblies (Fig. 2*B*).

Next, we tested the seeding activity of these fractions using biosensor cells. We found no seeding activity up to 3 weeks. We detected low seeding activity at 4 weeks, which increased dramatically at 5 to 6 weeks. At 4 weeks, monomer and smaller size oligomers (n = 1–3) accounted for most of the seeding, whereas by 5 weeks and beyond the seeding distributed across a range of assembly sizes (Fig. 2*C*). These results established that the very first seed-competent forms of tau occur as a monomer and very small assemblies, whereas larger, detergent-insoluble forms of protein appear several weeks later. By the end stage of disease, the seeding activity in all fractions was very high due to the prevalence of tau pathology.

### M_i_ and M_s_ have similar posttranslational modifications

**Figure 3:**
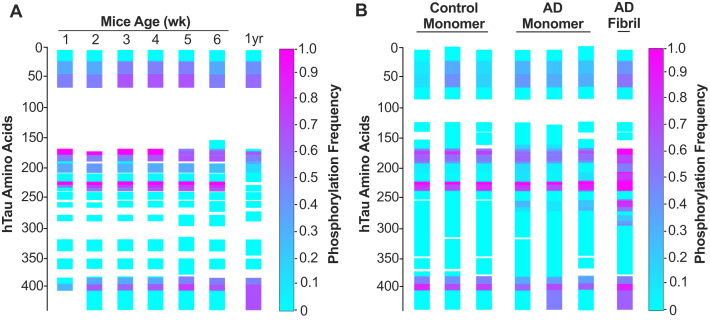
**Phosphorylation patterns on tau monomer isolated fromPS19 mice, AD, and age-matched controls.***A*, tau monomer isolated from PS19 mouse brains aged 1 to 6 weeks (n = 3 animals per week) was analyzed by mass spectrometry to determine phosphorylation patterns. Intensities of modified and unmodified peptides were compared to calculate the abundance of the modification. Phosphorylation frequency is coded from 0% (*cyan*) to 100% (*magenta*). *B*, similar analysis was performed on tau monomer samples isolated from three age-matched controls and three subjects with AD, and an AD fibril preparation, with human peptides mapped against 2N4R tau sequence. The gap in PTM seen in mice reflects the 1N4R tau isoform. Details of mass spectrometry are provided in Supporting Information Table and Figs. S3.1–S4.

It is unknown what initiates the conversion of M_i_ to M_s_, or what might maintain M_s_ in a seed-competent state. Tau phosphorylation was first noted in the setting of tau pathology (26, 27, 28) and has been thought to initiate aggregation. Hence, we examined the phosphorylation pattern of tau monomer isolated across mice aged 1 to 6 weeks and 1-year old, tau monomer purified from AD and control subjects, and arkosyl-insoluble tau from AD. After immunopurification, the monomer was isolated by SEC, denatured, proteolyzed with trypsin, and analyzed by mass spectrometry to determine posttranslational modifications. We observed a relatively conserved pattern of tau phosphorylation in week 1 to week 6 of mice, and some deviation in the 1-year-old mice (Figs. 3*A*, S3-1*A* and S3-2*A*). However, we observed no phosphorylation changes that explained the rapid increase in M_s_ seeding activity from week 3 to week 6.

We performed similar analyses on tau monomer isolated from human AD and control brains, as well as tau fibrils extracted from AD. We observed phosphorylation patterns in control and AD monomer samples similar to mice (Figs. 3*B* and S3-1*B*). We observed higher phosphorylation levels in both the number of modification sites and their frequencies in the insoluble AD tau fraction (Figs. 3*B*, S3-1*B* and S3-2*B*). In human samples we observed no phospho-residues that differentiated AD from the control monomer, except for S262, which was modified in 4% to 20% of peptides across the three AD samples but not in controls (Fig. S3-2*B*). S262 was modified in AD tau fibrils at ∼60% frequency (Fig. S3-2*B*). Human tau monomer was highly phosphorylated in all samples at T231, with control monomer positive at 65% to 71%, AD monomer positive at 82% to 94%, and ∼100% of AD fibrils positive (Fig. S3-2*B*). Overall, we observed no PTM patterns that differentiated AD *versus* control monomer.

We also measured tau ubiquitination and acetylation. We detected no modifications consistent with ubiquitination in tau monomer at any age of PS19 mice. We detected low levels of ubiquitination in tau monomer from human control and two of the AD brains (Fig. S4). K254 was ubiquitinated in all conditions at <1% frequency. Monomer from one AD brain exhibited three peptides ubiquitinated at 100% frequency (*i.e.* only ubiquitinated forms of these specific peptides were detected) (Fig. S3-3). However, ubiquitination frequencies of those residues were mostly <1% except for K257, with 71% ubiquitination. AD tau fibrils exhibited more ubiquitination sites and higher frequencies than the monomer (Fig. S3-3). We observed acetylation on K44 in tau monomer at 2 to 4 weeks in PS19 mice, but always at a very low frequency of 0.4% to 0.7% (not shown in figures but data available in Source Data File). Although peptide spanning residues 156 to 170 at 6 weeks showed 100% acetylation at K163, this peptide was not detected in samples of other ages, and therefore no clear conclusion can be derived for this position. In human tau monomer, we observed acetylation on K44 in all three controls and two-thirds of AD samples at a low frequency of 0.3% to 5%. AD fibrils had acetylation on K375 at 69% frequency (Fig. S3-4). Taken together, we found no correlation of ubiquitination or acetylation with seeding activity in tau monomer of PS19 mice, as ubiquitination was not detected at any age studied, while acetylation occurred at a very low level in both seed-positive and seed-negative conditions. In human samples, we observed several sites of acetylation and ubiquitination within the repeat domain region of AD fibrils. However, the occurrence of acetylation and ubiquitination on tau monomer was low, either from control or AD samples (with one exception of ubiquitination in AD3 monomer). Overall, we did not detect changes in phosphorylation, acetylation, or ubiquitination of M_s_ that explained its seeding activity.

## Discussion

Tauopathies are defined by fibrillar assemblies of tau protein. Our prior studies have defined two conformational states of tau monomer: inert (M_i_) and seed competent (M_s_). M_s_ self-assembles to form larger aggregates (16) and also encodes strains in biosensor cells (17). A critical unanswered question is whether M_s_ is derived from preexisting tau fibrils or whether it forms first as an initial step in pathogenesis and then leads to larger assemblies. We have previously determined that M_s_ typically exists in dynamic equilibrium *in vitro* with dimers and potentially trimers (20). We have now determined that M_s_ appears in the brain of a mouse model (in conjunction with dimers and trimers) within a defined time window, before larger assemblies have formed, and in the absence of detergent-insoluble deposits. We conclude that the first events in neurodegeneration, prior to the development of neurofibrillary tangles, comprise a shift in tau conformation from M_i_ to M_s_. At least in the PS19 mouse model, where we can conduct longitudinal studies, we suggest that disease onset (at least as pertains to tau seeding) can be defined by the first moment of conformational change from M_i_ to M_s_.

### No phosphorylation pattern distinguishes M_s_ and M_i_

Posttranslational modifications on insoluble tau, especially phosphorylation, acetylation, and ubiquitination, are well described in humans (29), such that phosphorylation at S202/T205 is used routinely to classify the extent of disease in humans (30) and mice (31) with the AT8 monoclonal antibody (23). This has led to the hypothesis that posttranslational modifications (PTMs) drive pathogenesis. Our findings do not support a primary role for tau PTM in disease initiation, at least in PS19 mice. When we resolved tau monomer over 1-week intervals in the PS19 mice, we observed no modifications that correlated with conversion of M_i_ to M_s_, despite an increase in seeding activity >50-fold in the 4- to 6-week time window. Furthermore, in mass spectrometry (MS) analyses of tau from human brains, AD monomer (which had seeding activity) more closely resembled that of control brain than insoluble tau from AD, which had many PTMs. MS reports on all tau modifications together and cannot determine whether a tiny seed-competent fraction of monomer has a specific PTM that is responsible for its activity. To partially address this question, we have tested whether tau monomer derived from PS19 mice (Fig. S4, *A* and *B*) or two different AD subjects (Fig. S4, *C* and *D*) retains its seeding activity after protein phosphatase PP2A treatment (32). We could not confirm whether every phospho-residue was removed, but we did not observe any loss of seeding activity following phosphatase treatment.

In summary, we cannot completely exclude that monomer seeding activity at 4 weeks results from a low abundance of distinctly modified peptide not detected by mass spectrometry. In addition, we have not accounted for any potential variance in the kinase/phosphatase activities during the developmental stages of mice. Nevertheless, the fact that monomer seeding at week 4 increased 20-fold by week 5, and 100-fold by week 6, with essentially no change in overall phosphorylation patterns, indicates that within the limits of detection (and in contrast to insoluble tau) we could not correlate tau M_s_ seeding with a distinct phosphorylation pattern.

### M_s_ precedes larger tau assemblies

In our prior work we have observed that M_s_ can be created by sonication of fibrils (16, 25) or by conversion of M_i_ with heparin exposure (16, 20). Work by the Nussbaum-Krammer laboratory indicates that chaperone treatment of fibrils *in vitro* liberates seed-competent monomer and possibly tau oligomers (18). Hence in humans and mice it has remained an open question as to which actually comes first: large assemblies *versus* M_s_, and whether M_s_ might derive from preexisting fibrillar species. This work answers this question as well as possible with current technology. We find that, in a very short period of time, over a 7-day window between weeks 3 and 4 in the PS19 mouse, M_i_ begins to convert from a state in which it is exclusively monomer to a seed-competent form (with associated dimers and trimers) but very few larger species. Yet by 5 to 6 weeks, a full range of assembly sizes is detected. This is about 2 to 6 weeks before the appearance of detergent-insoluble (large) tau assemblies, which we detected by seeding activity at 2 months and by Western blot at 3 months. The simplest interpretation of these results is that seed-competent tau monomer, M_s_, is formed before subsequent oligomers and fibrillar species and does not derive from them. Although not directly addressed here, this observation calls into question the idea that seeds form only after high local concentrations of tau have occurred. Instead, we favor a model in which specific events, potentially ligand binding, facilitate conversion of tau from one conformational state (M_i_) to another (M_s_), prior to formation of larger, more stable assemblies.

### M_i_ to M_s_ conversion: The first detectable initiation of tau pathology *in vivo*

The goal of any mechanism-based therapy for tauopathy will be to intervene before disease onset. Hence a major challenge has been to define the point in human disease when this occurs. We still do not know the answer to this question in humans, but our demarcation of a 7-day period between weeks 3 and 4 in the life of a PS19 mouse, during which we can detect the first changes in tau conformation, indicates that M_s_ formation precedes all subsequent neuropathology. Hence, we propose that the biochemical initiation of disease occurs at this moment and that understanding mechanisms that underlie this process will reveal the basis of neurodegeneration.

## Experimental procedures

### Animal maintenance

PS19 mice expressing 4R1N P301S human tau under the murine prion promoter were used (22). All mice involved in this study were housed under a 12-h light/dark cycle and were provided food and water *ad libitum*. All experiments involving animals were approved by the University of Texas Southwestern Medical Center Institutional Animal Care and Use Committee (IACUC).

### Isolation of mouse brain

Animals were anesthetized with isoflurane and perfused with cold PBS. Brains were hemidissected. The right hemisphere was frozen in liquid nitrogen and stored at −80 °C for subsequent biochemical assays while the left hemispheres were drop-fixed in Phosphate-Buffered 4% Paraformaldehyde (FD NeuroTechnologies) overnight at 4 °C. Left hemispheres were then placed in 10% sucrose in PBS for 24 h at 4 °C, followed by 24 h in 20% sucrose in PBS at 4 °C, and finally stored in 30% sucrose in PBS at 4 °C until sectioning.

### Immunohistochemistry of mouse tissue

A sliding-base freezing microtome (Thermo Fisher Scientific) was used to collect 40 μm free-floating sagittal sections from the fixed mouse brains. Sections were stored in cryoprotectant at 4 °C until immunohistochemistry was performed. Slices were first blocked for 1 h with 5% bovine serum albumin in Tris-buffered saline (TBS) with 0.25% Triton X-100 (blocking buffer). Brain slices were incubated with biotinylated AT8 antibody (1:500, Thermo Scientific) overnight in blocking buffer at 4 °C. Slices were subsequently incubated with the VECTASTAIN Elite ABC Kit (Vector Labs) in TBS prepared according to the manufacturer’s protocol for 30 min, followed by DAB development using the DAB Peroxidase Substrate Kit (Vector Labs). Slices were imaged using the Olympus Nanozoomer 2.0-HT (Hamamatsu) in the University of Texas Southwestern Medical Center Whole Brain Microscopy Core Facility, RRID:SCR_017949.

### Tau extraction from mouse/human brain and characterization by SEC

Right hemispheres of three PS19 mice brains of identical ages ranging from 1 to 6 weeks and 1 year old were gently homogenized in 1:10 ratio w/v of TBS buffer containing protease inhibitor cocktails (Roche) using a Dounce homogenizer. Samples were centrifuged at 21,000*g* for 15 min at 4 °C to remove cellular debris. The supernatant was loaded onto a Superdex 200 Increase 10/300 GL column (GE Healthcare) and fractions containing monomer (B5), dimer (B7), trimer (B8), 10-mer (A7), and 20-mer (A4) were isolated according to prior methods based on molecular reference standards (25), and then partitioned into aliquots, snap frozen, and stored at −80 °C for seeding assays and Western blots.

For the PTM studies, left hemispheres of three mouse brains of identical ages were Dounce homogenized and centrifuged at 21,000*g*. The supernatant was collected and incubated with 400 μg of monoclonal anti-tau antibody (HJ8.5) and 2 ml of Pierce ProteinA/G magnetic beads (ThermoFisher) overnight. The beads were washed three times and eluted with a low pH buffer and immediately neutralized. The eluted samples were run on a Superdex 200 (10/300) column (GE) to fractionate different tau species including monomer, dimer, trimer, 10-mer, and 20-mer. Monomer fraction was concentrated by 5KMWC filter (Pierce), snap frozen, and used for mass spectrometry studies of PTMs.

To extract insoluble Tau fibril from mouse brain it was Dounce homogenized in PHF buffer (10 mM Tris-HCl (pH 7.4), 0.8 M NaCl, 1 mM EDTA, protease, and phosphatase inhibitor) at a 1:10 ratio (w/v). The homogenate was then centrifuged at 21,000*g* and 1% sarkosyl was added to the supernatant followed by end-to-end rotation at room temperature for 1 h. It was then centrifuged at 186,000*g* for 1 h, and the pellet was washed with PBS followed by a second round of ultracentrifugation at 186,000*g*. The supernatant was discarded, and the pellet was resuspended in 1 ml PBS to be analyzed by seeding assay and Western blot.

Tau extraction from human brains was adopted from (16) and is similar to the procedure for mouse brains. One gram of frontal lobe sections from subjects with AD at late Braak stage (VI) and age-matched controls lacking evident tau pathology wase gently homogenized at 4 °C in 10 ml of TBS buffer containing protease inhibitor cocktails (Roche) using a Dounce homogenizer. Samples were centrifuged at 21,000*g* for 15 min at 4 °C to remove cellular debris. The supernatant was partitioned into aliquots, snap frozen, and stored at −80 °C. Immunopurification was performed with HJ8.5 anti-tau antibody (33) at a ratio of 1:50 (1 μg mAb per 50 μg of total protein), incubating overnight at 4 °C while rotating. Beads were washed with TBS buffer before overnight incubation at 4 °C. The complexes were centrifuged at 1000*g* for 3 min, and the supernatant was discarded. The beads were washed with Pierce Gentle Ag/Ab Binding Buffer, pH 8.0 (ThermoFisher) three times. Tau was eluted from the beads using 180 μl low pH elution buffer (ThermoFisher), incubated at room temperature for 10 min, followed by neutralization with 18 μl Tris-base pH 8.5. The residual beads were removed by magnet, and the supernatant was loaded onto a Superdex 200 Increase 10/300 GL column (GE Healthcare). SEC fractions were flash frozen and stored at −80 °C.

### Western and dot blot

For Western blot and dot blot analyses, immunoprecipitation was eliminated to maximize the quantity of tau protein and enhance tau visibility on the blots. SEC fractions were boiled for 5 min with SDS-PAGE sample buffer and loaded into a NuPAGE 4% to 12% Bis-Tris Gel in a chamber filled with NuPAGE MOPS SDS running buffer (ThermoFisher) and run at 100 V for ∼110 min. Samples were then transferred to a PVDF membrane using a semidry transfer apparatus (Bio-Rad). After being blocked in 5% milk (Bio-Rad), the membrane was incubated with primary polyclonal rabbit anti-human tau antibody (Dako, Agilent) at 1:2000 on a shaker overnight at 4 °C. It was then washed with TBST 3 times for 30 min and was incubated with anti-rabbit secondary antibody for 1 h at room temperature. The membrane was then washed with TBST on a shaker for 10 min twice, before the final rinse with TBS. The membrane was then developed with ECL prime Western blot detection kit (GE Lifescience) for 3 min before being imaged by a digital imager (Syngene).

### Liposome-mediated transduction of tau seeds

Stable cell lines were plated at a density of 35,000 cells per well in a 96-well plate. Eighteen hours later, at 50% confluency, cells were transduced with proteopathic seeds. Insoluble tau extracted from mice hemibrains was diluted in PBS to the final ratio of 1:200 of a hemibrain homogenate volume (5 μl insoluble tau extract + 5 μl PBS per each well of a 96-well plate). This volume was selected based on titrating insoluble tau extract of the 6-month-old mice (data not shown), which demonstrated the highest seeding activity without causing toxicity on biosensor cells. Transduction complexes were made by combining [8.75 μl Opti-MEM (Gibco) +1.25 μl Lipofectamine 2000 (Invitrogen)] with [Opti-MEM + proteopathic seeds] for a total volume of 20 μl per well. Liposome preparations were incubated at room temperature for 20 min before adding to cells. Cells were incubated with transduction complexes for 48 h.

### FRET flow cytometry

Cells were harvested with 0.05% trypsin and then fixed in 2% paraformaldehyde (Electron Microscopy Services) for 10 min, then resuspended in flow cytometry buffer. An LSRFortessa (BD Biosciences) was used to perform FRET flow cytometry. To measure cyan fluorescent protein emission and FRET, cells were excited with the 405-nm laser, and fluorescence was captured with a 405/50 nm and 525/50 nm filter, respectively. To measure YFP, cells were excited with a 488 laser and fluorescence was captured with a 525/50-nm filter. The integrated FRET density, defined as the percentage of FRET-positive cells multiplied by the median fluorescence intensity of FRET-positive cells, was used for all analyses (24, 34). For each experiment, 20,000 cells per replicate were analyzed and each condition was analyzed in triplicate. Data analysis was performed using FlowJo v10 software (Treestar).

### Sample preparation for mass spectrometry and analysis of PTMs

The sarkosyl insoluble sample as well as the concentrated soluble monomer fractions were denatured in 8 M urea and reduced with 2.5 mM TCEP at 37 °C, 600 RPM for 30 min. The sample was then alkylated with 5 mM iodoacetamide for 30 min at RT and protected from light. The sample solutions were diluted to 1 M urea with 50 mM ammonium hydrogen carbonate, and trypsin (Promega) was added at an enzyme-to-substrate ratio of 1:50. Proteolysis was carried out at 37 °C overnight followed by acidification with formic acid to 2% (v/v). Samples were then purified by solid-phase extraction using Sep-Pak tC18 cartridges (Waters), dried, and the resulting peptides were reconstituted in 10 μl of 2% (v/v) acetonitrile (ACN) and 0.1% trifluoroacetic acid in water. A portion of the sample was injected onto an Orbitrap Fusion Lumos mass spectrometer (Thermo Electron) coupled to an Ultimate 3000 RSLC-Nano liquid chromatography systems (Dionex). Samples were injected onto a 75-μm i.d., 75-cm long EasySpray column (Thermo), and eluted with a gradient from 0% to 28% buffer B over 90 min. Buffer A contained 2% (v/v) ACN and 0.1% formic acid in water, and buffer B contained 80% (v/v) ACN, 10% (v/v) trifluoroethanol, and 0.1% formic acid in water. The mass spectrometer operated in positive ion mode. MS scans were acquired at 120,000 resolution in the Orbitrap, and up to ten MS/MS spectra were obtained in the ion trap for each full spectrum acquired using higher-energy collisional dissociation for ions with charges 2 to 7. Dynamic exclusion was set for 25 s after an ion was selected for fragmentation. Raw MS data files were analyzed using Proteome Discoverer v2.2 (Thermo), with peptide identification performed using Sequest HT searching against the human or mouse protein database from UniProt (downloaded on January 8, 2021) including the tau isoform of interest (with a total of 75,552 entries searched). Fragment and precursor tolerances of 10 ppm and 0.6 Da were specified, and three missed cleavages were allowed. Carbamidomethylation of Cys was set as a fixed modification, with oxidation of Met and phosphorylation of Ser, Thr, and Tyr set as variable modifications. For identification of acetylation and ubiquitination, Lys and Met were set as a variable modifications with fixed modifications on Cys. Modification frequency per position was calculated as the ratio of modified peptide abundance over the sum of all peptide abundances containing the specific residue. The false-discovery rate cutoff was 1% for all peptides. Phosphorylation, acetylation, and ubiquitination posttranslational searches were carried out independently from the same original mass spectrometry datasets.

### Recombinant PP2A production

Genes encoding the full-length sequences of Human PP2A A subunit and PP2A C subunit with N-terminal His tag and noncleavable HA tag were subcloned into the pFastBac-Dual vector (Invitrogen). Sequences of PP2A A and C subunits are shown in Table 1, with 10xHIS/TEV protease cleave site/HA tag indicated (underlined). The plasmid containing PP2A A and C subunits was transformed into DH10Bac *Escherichia coli* for transposition into the bacmid. The purified bacmid with Cellfectin II reagent was transfected into Sf9 cells grown in media SF-900 III SFM (Gibco) supplemented with 10% fetal bovine serum incubated at 27 °C with 130 rpm for 7 days. P1 virus was spun down at 1500 rpm for 5 min, and high-titer P2 virus stock was produced from the P1 virus (1:200 v/v infection). High Five insect cells for the expression of PP2A A/C complex were cultured in EX-CELL 405 Serum-Free medium (Millipore-Sigma) at 27 °C with 130 rpm. Cells were harvested 2 days after viral infection (1:50 v/v) and resuspended in 50 mM Tris-HCl, 100 mM NaCl, 2 mM MgCl_2_, 5 mM βME, and 20 mM imidazole, pH 8. The lysate was homogenized by GEA Niro Soavi’s PandaPLUS 2000, followed by 15,000*g* spin. The supernatant was incubated with Ni-NTA beads with head-to-head rotation at 4 °C for 1 h. The Ni-NTA mix was pelleted at 1000*g*, 4 °C for 5 min and the pellet resuspended in lysis buffer and loaded onto a gravity column. The bead bed was washed by 40 column volumes of 50 mM Tris-HCl, 100 mM NaCl, 2 mM MgCl_2_, 5 mM βME, and 50 mM imidazole, pH 8. Although only the PP2A C subunit has His-tag, the high binding affinity of the PP2A A and C subunits allows coelution of the complex, which was eluted in 50 mM Tris-HCl, 100 mM NaCl, 2 mM MgCl_2_, 5 mM βME, and 250 mM imidazole, pH 8. The Ni elution was buffer exchanged into 20 mM Tris-HCl, 50 mM NaCl, 2 mM MgCl_2_, and 1 mM DTT, pH 8, by PD-10 column (GE). The exchanged fractions were applied to a HiTrap Q HP (GE) and eluted with a 50 mM to 1 M NaCl gradient. Fractions that contain both A and C subunits were collected, aliquoted, and flash frozen.

**Table 1:** PP2A subunit amino acid sequences

PP2A subunit	Amino acid sequence
A	MAAADGDDSLYPIAVLIDELRNEDVQLRLNSIKKLSTIALALGVERTRSELLPFLTDTIYDEDEVLLALAEQLGTFTTLVGGPEYVHCLLPPLESLATVEETVVRDKAVESLRAISHEHSPSDLEAHFVPLVKRLAGGDWFTSRTSACGLFSVCYPRVSSAVKAELRQYFRNLCSDDTPMVRRAAASKLGEFAKVLELDNVKSEIIPMFSNLASDEQDSVRLLAVEACVNIAQLLPQEDLEALVMPTLRQAAEDKSWRVRYMVADKFTELQKAVGPEITKTDLVPAFQNLMKDCEAEVRAAASHKVKEFCENLSADCRENVIMSQILPCIKELVSDANQHVKSALASVIMGLSPILGKDNTIEHLLPLFLAQLKDECPEVRLNIISNLDCVNEVIGIRQLSQSLLPAIVELAEDAKWRVRLAIIEYMPLLAGQLGVEFFDEKLNSLCMAWLVDHVYAIREAATSNLKKLVEKFGKEWAHATIIPKVLAMSGDPNYLHRMTTLFCINVLSEVCGQDITTKHMLPTVLRMAGDPVANVRFNVAKSLQKIGPILDNSTLQSEVKPILEKLTQDQDVDVKYFAQEALTVLSLA
C	MHHHHHHHHHHDYDIPTTENLYFQGYPYDVPDYAMDEKVFTKELDQWIEQLNECKQLSESQVKSLCEKAKEILTKESNVQEVRCPVTVCGDVHGQFHDLMELFRIGGKSPDTNYLFMGDYVDRGYYSVETVTLLVALKVRYRERITILRGNHESRQITQVYGFYDECLRKYGNANVWKYFTDLFDYLPLTALVDGQIFCLHGGLSPSIDTLDHIRALDRLQEVPHEGPMCDLLWSDPDDRGGWGISPRGAGYTFGQDISETFNHANGLTLVSRAHQLVMEGYNWCHDRNVVTIFSAPNYCYRCGNQAAIMELDDTLKYSFLQFDPAPRRGEPHVTRRTPDYFL

Underlined amino acids are cleaved by TEV protease.

### PP2A dephosphorylation of mouse and human brain isolated tau monomer

Tau monomer from 1-year-old P301S mouse and human AD brain was extracted using the same protocol as described above except for the elimination of immunoprecipitation before SEC and 2-fold higher concentrating for the SEC fraction, which were to enrich tau for the downstream Western blot analysis. A volume of 50 μl of tau monomer from either mouse or human brain was incubated with 50 μl of either 10 μM PP2A A+C complex or buffer (20 mM Tris, 100 mM NaCl, 1 mM DTT, pH 8) for 3 h at 37 °C. Immunoprecipitation with 50 μl of magnetic Dynabeads Protein A (ThermoFisher) was used for each condition to extract tau from the mixture. The beads were first incubated with 5 μg of HJ8.5 antibody for 40 min followed by a 200 μl PBST wash. A volume of 100 μl of the experiment mixture was diluted 2-fold then incubated with the beads–antibody conjugate for 1.5 h. The beads–antibody–tau conjugate was washed three times with 200 μl Pierce Gentle Ag/Ab Binding Buffer, pH 8.0 (ThermoFisher), after which it was eluted with 45 μl Pierce IgG Elution Buffer (ThermoFisher) for 5 min followed by neutralization with 4.5 μl Tris-base pH 8.5. The elution was split for seeding assay and Western blots. Seeding assay was conducted following the protocol described above and quantified by FRET flow cytometry. Conditions with or without PP2A were analyzed by SDS-PAGE Western blot with rabbit polyclonal anti-tau (Dako, Agilent) to show that the amount of tau after immunoprecipitation was comparable between the two conditions. Zn^2+^ SuperSep Phos-tag (50 μmol/l) 12.5% acrylamide SDS-PAGE (Fujifilm) was used to demonstrate PP2A dephosphorylation efficacy on tau. The phos-tag Western blot was used similarly to conventional SDS-PAGE Western blot, except for first the use of a different running buffer (25 mM Tris, 192 mM glycine, 0.1% (w/v) SDS), the use of a special EDTA-free ladder WIDE-VIEW Prestained Protein Size Marker Ⅲ (Fujifilm), and an additional step of three washes with 10 mmol/l EDTA in transfer buffer before the transfer.

## Data availability

The mass spectrometry data have been deposited in MassiveIVE (https://massive.ucsd.edu/ProteoSAFe/dataset.jsp?task=ff7c769df6f54076be7a8135f4145cdf). An in-house Python script was created (Github link: https://git.biohpc.swmed.edu/s184069/abundanceparser) to parse the relative abundance (in percentage) of the phosphorylated, acetylated, and ubiquitinated peptides to the total abundance for each peptide sequence detected. An in-house MATLAB script was generated to illustrate the relative abundance of phosphorylated, acetylated, and ubiquitinated peptides compared (blue to magenta) with unmodified peptides (cyan) with color coding on a sequence map. Each search was performed independently. Description of mass spectrometry files for AD and control cases and description of mass spectrometry for mouse samples are provided in the Source Data File.

## Supporting information

This article contains supporting information.

## Conflict of interest

The authors declare that they have no conflicts of interest with the contents of this article.

## Supporting information

Supplemental Figure S1 **A Superdex 200 column was used to fractionate brains.***A*, chromatogram of size standards used (Biorad). *B*, chromatograms of total brain lysates from PS19 mice at the indicated ages, with fractions corresponding with predicted assembly sizes (n = 1,2,3…) indicated by *arrows*. Absorbance is reflected in arbitrary units (A.U.).

Supplemental Figure S2 **Full Western blot image of cropped gel in**Figure 2.

Supplemental Figure S3-1 **Phosphorylation frequency and peptide coverage for PS19, control monomer, AD tau monomer and AD fibril.***A*, peptide coverage map for tau monomer isolated from PS19 mice 1 to 6 weeks and 1 year. Peptide fragments are colored by phosphorylation frequency from 0 (*cyan*) to 100% (*magenta*). *B*, peptide coverage map for tau monomer isolated from age-matched controls, AD and AD fibrils. Peptide fragments are colored by phosphorylation frequency from 0 (*cyan*) to 100% (*magenta*). Each bar represents a different peptide detected in the search, offset to allow discrimination. The search for tau peptides with and without phosphorylation post-translational modifications was carried out independently using the original mass spectrometry data.

Supplemental Figure S3-2 **Cumulative phosphorylation frequency by amino acid position.***A*, cumulative frequency of phosphorylation for specific sites across different peptide sequences for PS19 tau monomer samples isolated from mice aged 1 to 6 weeks and 1 year. *B*, cumulative frequency of phosphorylation for specific sites across different peptide sequences for tau monomer isolated from age-matched controls, AD and AD fibril.

Supplemental Figure S3-3 **Ubiquitination patterns in AD and control tau samples.***A*, peptide coverage map for tau monomer isolated from age-matched controls, AD and AD fibrils. Peptide fragments are colored by acetylation frequency from 0 (*cyan*) to 100% (*magenta*). *B*, cumulative frequency of ubiquitination for specific sites across different peptide sequences for AD3 tau monomer (the only sample with ubiquitination) and AD fibrils. Each bar represents a different peptide detected in the search, offset to allow discrimination. The search for tau peptides with and without ubiquitination post-translational modifications was carried out independently using the original mass spectrometry data.

Supplemental Figure S3-4 **Acetylation patterns for tau monomer isolated from PS19 mouse and human control and AD samples.***A*, peptide coverage map for tau monomer isolated from PS19 mice 1 to 6 weeks and 1 year. Peptide fragments are colored by acetylation frequency from 0 (*cyan*) to 100% (*magenta*). *B*, peptide coverage map for tau monomer isolated from age-matched controls, AD and AD fibrils. Peptide fragments are colored by acetylation frequency from 0 (*cyan*) to 100% (*magenta*). *C*, cumulative frequency of acetylation for specific sites across different peptide sequences for AD fibrils. Each bar represents a different peptide detected in the search, offset to allow discrimination. The search for tau peptides with and without acetylation post-translation modifications was carried out independently using the original mass spectrometry data.

Supplemental Figure S4 **Seeding activity of dephosphorylated tau monomer from AD and mouse.***A*, Western blot analysis of tau monomer isolated from PS19 mice resolved by SDS-PAGE and Phos-tag SDS-PAGE probed with anti-tau antibodies reveal patterns consistent with tau phosphorylation at multiple sites. Treatment of samples with phosphatase collapses bands on Phos-tag gel, indicating dephosphorylation. *B*, four independent replicates of tau monomer extracted from 1 year old PS19 mouse brains. Samples were treated with PP2A phosphatase (*dark grey*) and compared to non-treated control samples (*grey*) in a tau seeding assay. Error bars = S.D. *C*, Western blot analysis of tau monomer isolated from AD brains and resolved by SDS-PAGE and Phos-tag SDS-PAGE probed with anti-tau antibody. Treatment of samples with phosphatase collapses bands on Phos-tag gel, indicating dephosphorylation. *D*, four independent replicates of tau monomer were extracted from two different AD brains. Samples were treated with PP2A phosphatase (*dark grey*) and compared to non-treated samples (*grey*) in a tau seeding assay. Error bars = S.D.

Source Data File Tabs within this Excel file show the following: Mouse Brain Sample IDs; Human Brain Sample IDs; Mouse ages 1 to 6 week monomer PTMs; Mouse age 1 year PTM; Human Monomer PTM; Human Fibril PTM.
