# Supplementary figures and images for: Seed-competent tau monomer initiates pathology in a tauopathy mouse model
Source: J Biol Chem. 2022 Jun 22;298(8):102163. doi: 10.1016/j.jbc.2022.102163 (PMC9307951; doi:10.1016/j.jbc.2022.102163)

# A

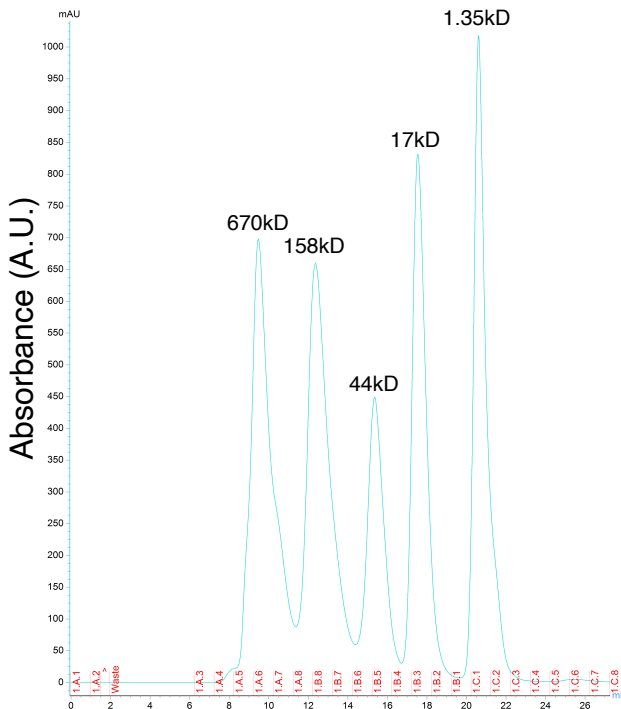

# B

# B

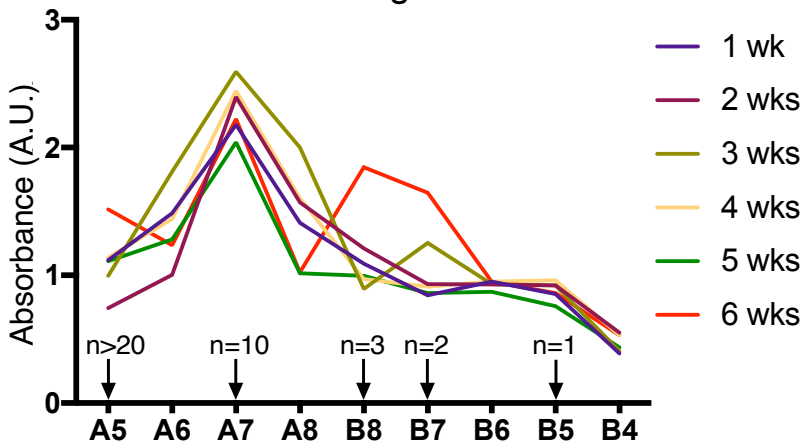

Supplement: Supplemental Figure S1 — A Superdex 200 column was used to fractionate brains.A, chromatogram of size standards used (Biorad). B, chromatograms of total brain lysates from PS19 mice at the indicated ages, with fractions corresponding with predicted assembly sizes (n = 1,2,3…) indicated by arrows. Absorbance is reflected in arbitrary units (A.U.). [file mmc1.pdf]

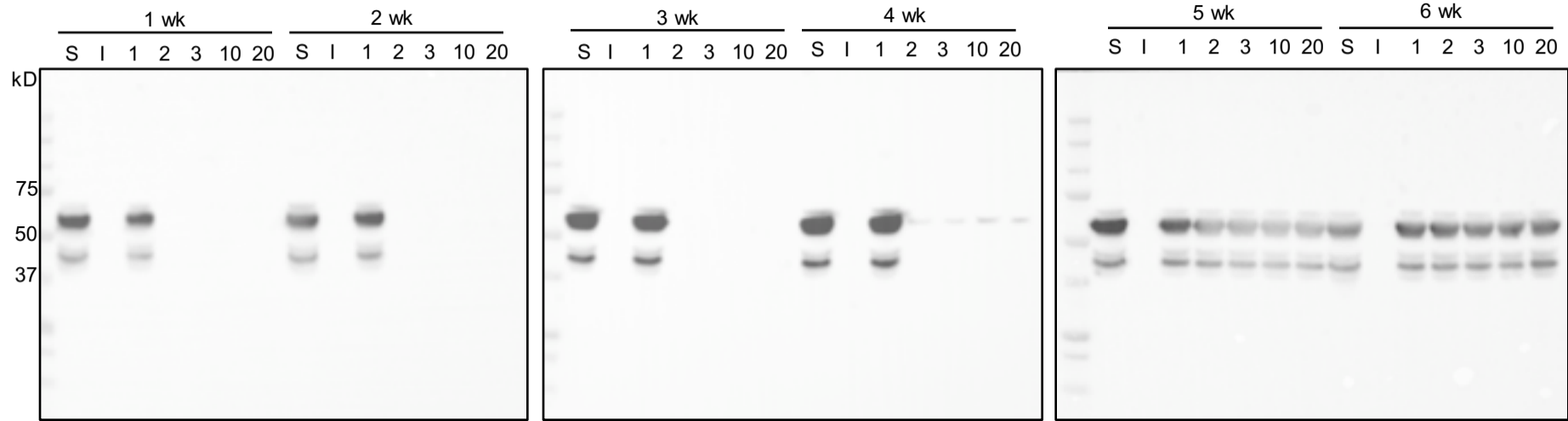

Supplement: Supplemental Figure S2 — Full Western blot image of cropped gel inFigure 2. [file mmc2.pdf]

**A**

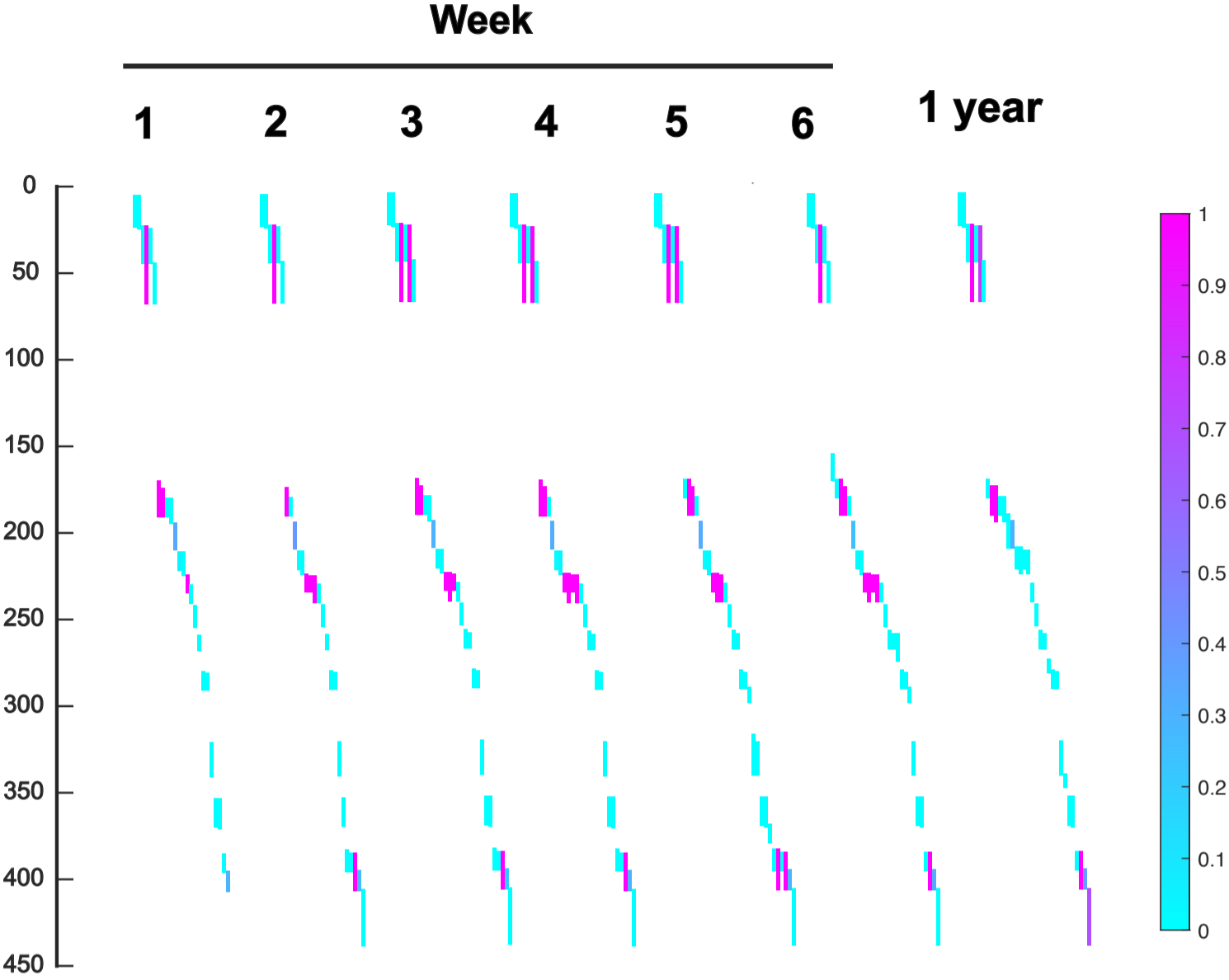

**B**

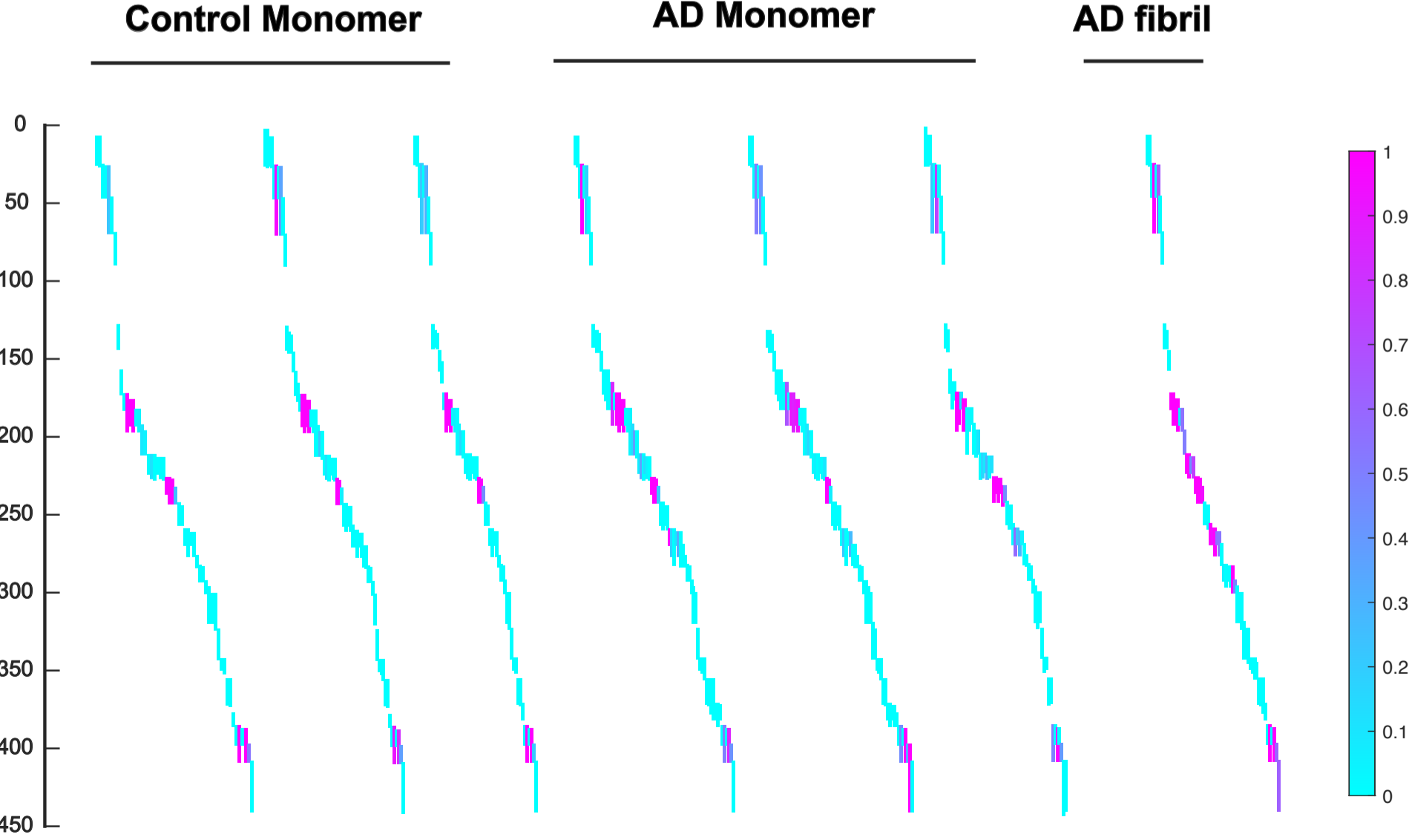

Supplement: Supplemental Figure S3-1 — Phosphorylation frequency and peptide coverage for PS19, control monomer, AD tau monomer and AD fibril.A, peptide coverage map for tau monomer isolated from PS19 mice 1 to 6 weeks and 1 year. Peptide fragments are colored by phosphorylation frequency from 0 (cyan) to 100% (magenta). B, peptide coverage map for tau monomer isolated from age-matched controls, AD and AD fibrils. Peptide fragments are colored by phosphorylation frequency from 0 (cyan) to 100% (magenta). Each bar represents a different peptide detected in the search, offset to allow discrimination. The search for tau peptides with and without phosphorylation post-translational modifications was carried out independently using the original mass spectrometry data. [file mmc3.pdf]

**A**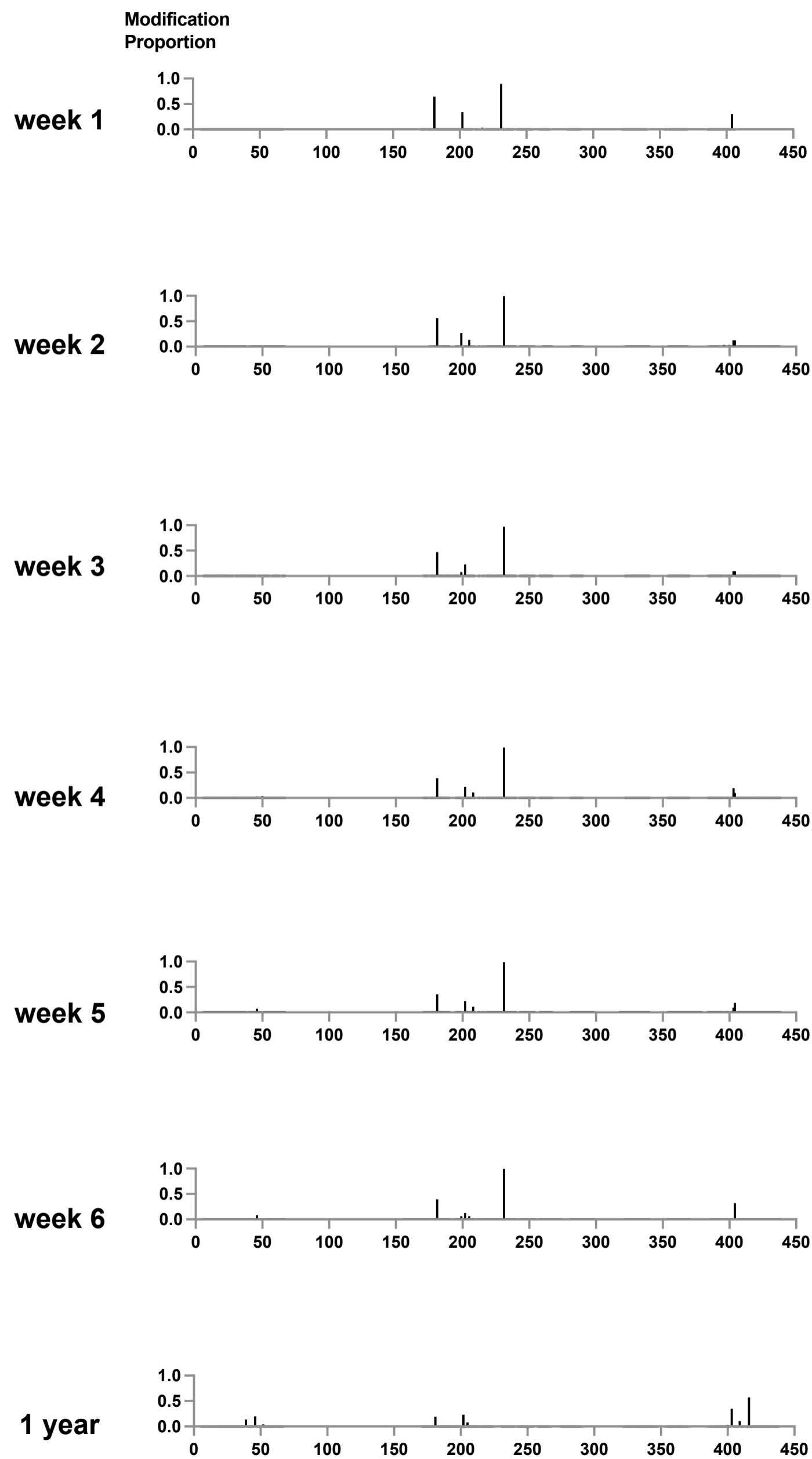**B**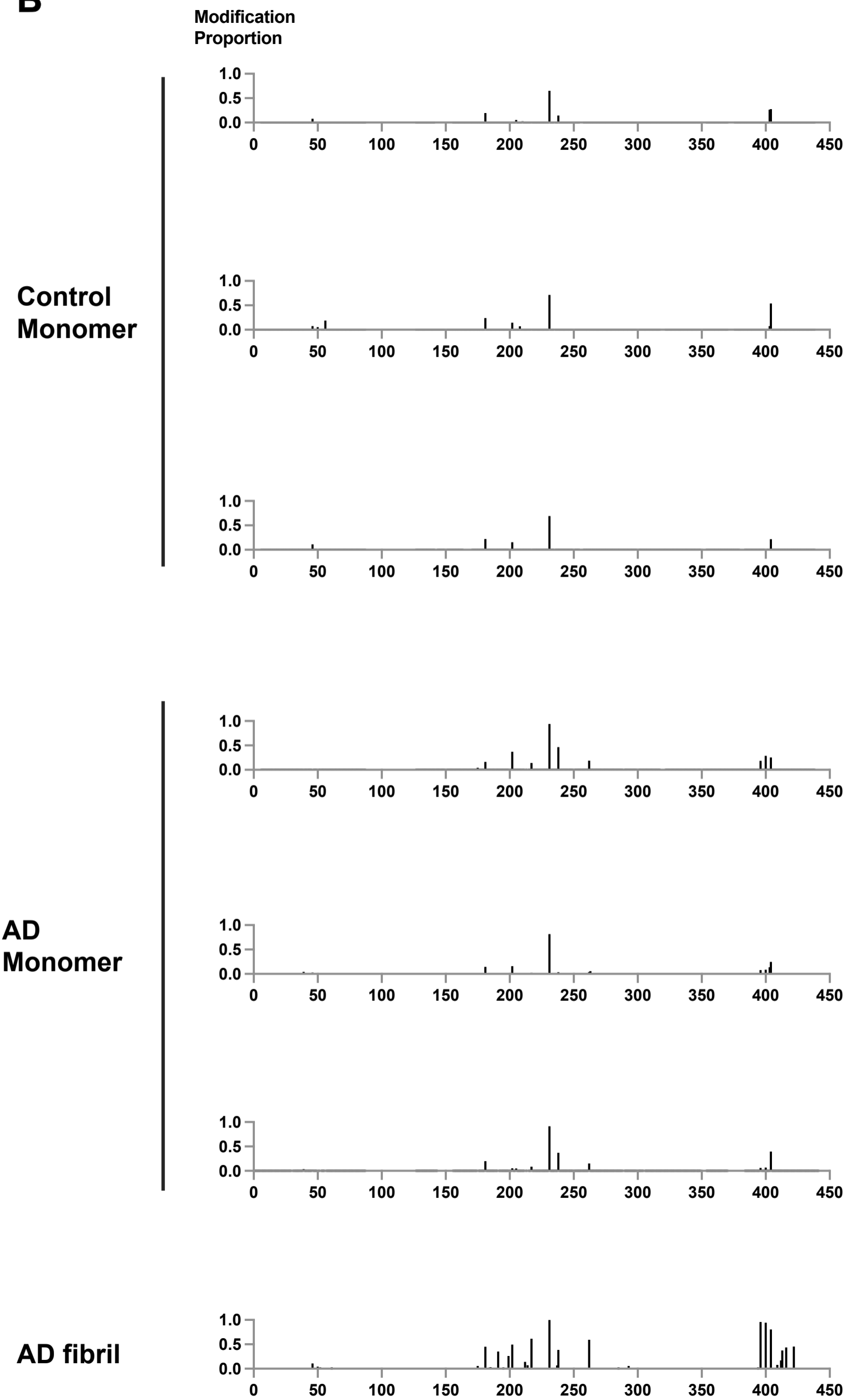

Supplement: Supplemental Figure S3-2 — Cumulative phosphorylation frequency by amino acid position.A, cumulative frequency of phosphorylation for specific sites across different peptide sequences for PS19 tau monomer samples isolated from mice aged 1 to 6 weeks and 1 year. B, cumulative frequency of phosphorylation for specific sites across different peptide sequences for tau monomer isolated from age-matched controls, AD and AD fibril. [file mmc4.pdf]

**A**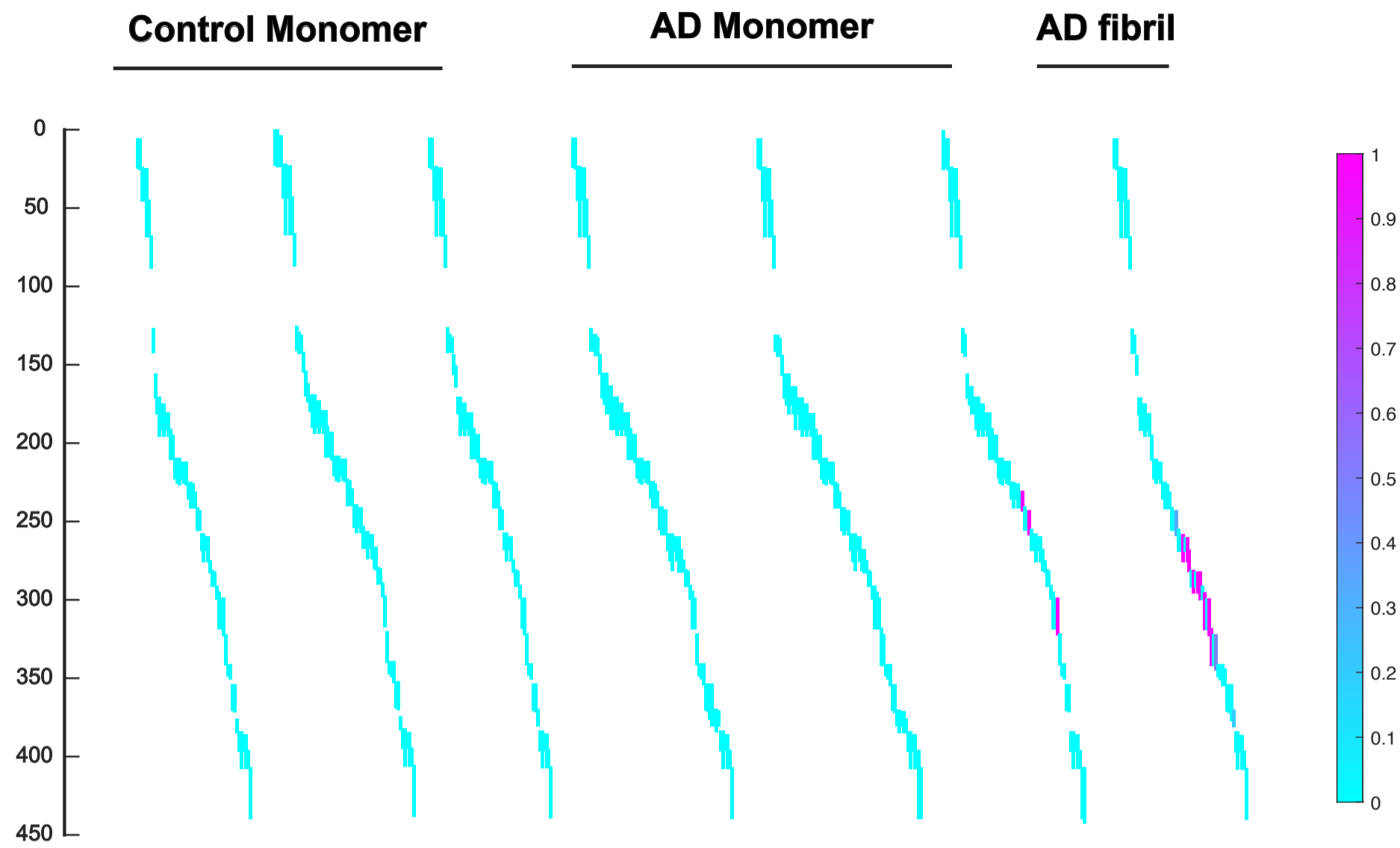**B**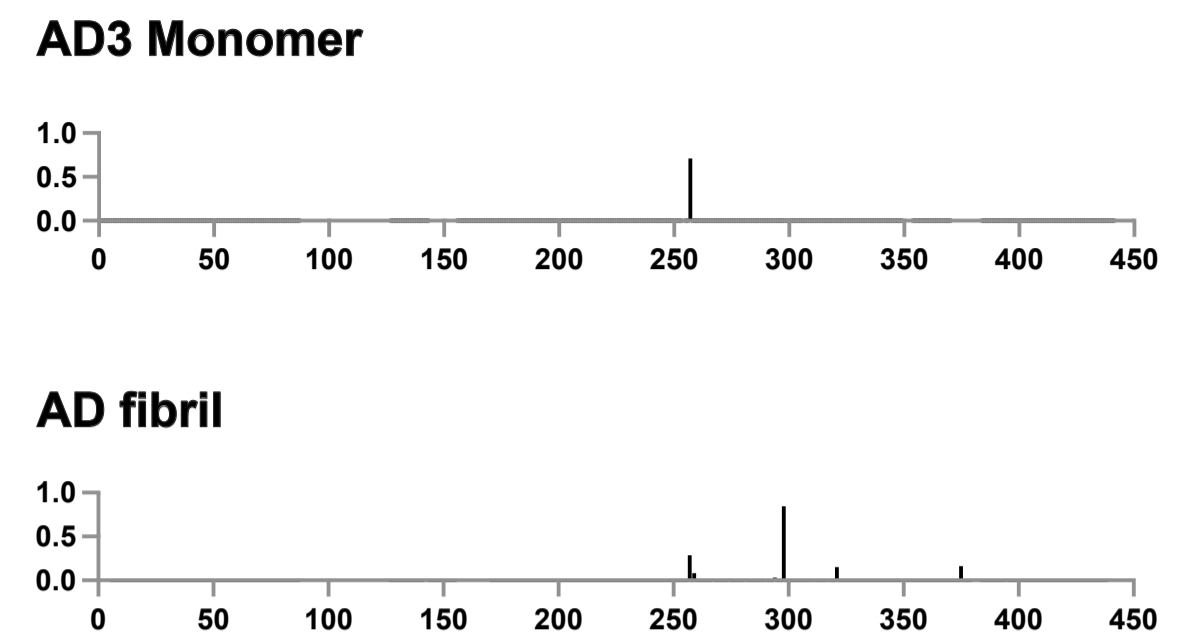

Supplement: Supplemental Figure S3-3 — Ubiquitination patterns in AD and control tau samples.A, peptide coverage map for tau monomer isolated from age-matched controls, AD and AD fibrils. Peptide fragments are colored by acetylation frequency from 0 (cyan) to 100% (magenta). B, cumulative frequency of ubiquitination for specific sites across different peptide sequences for AD3 tau monomer (the only sample with ubiquitination) and AD fibrils. Each bar represents a different peptide detected in the search, offset to allow discrimination. The search for tau peptides with and without ubiquitination post-translational modifications was carried out independently using the original mass spectrometry data. [file mmc5.pdf]

**A**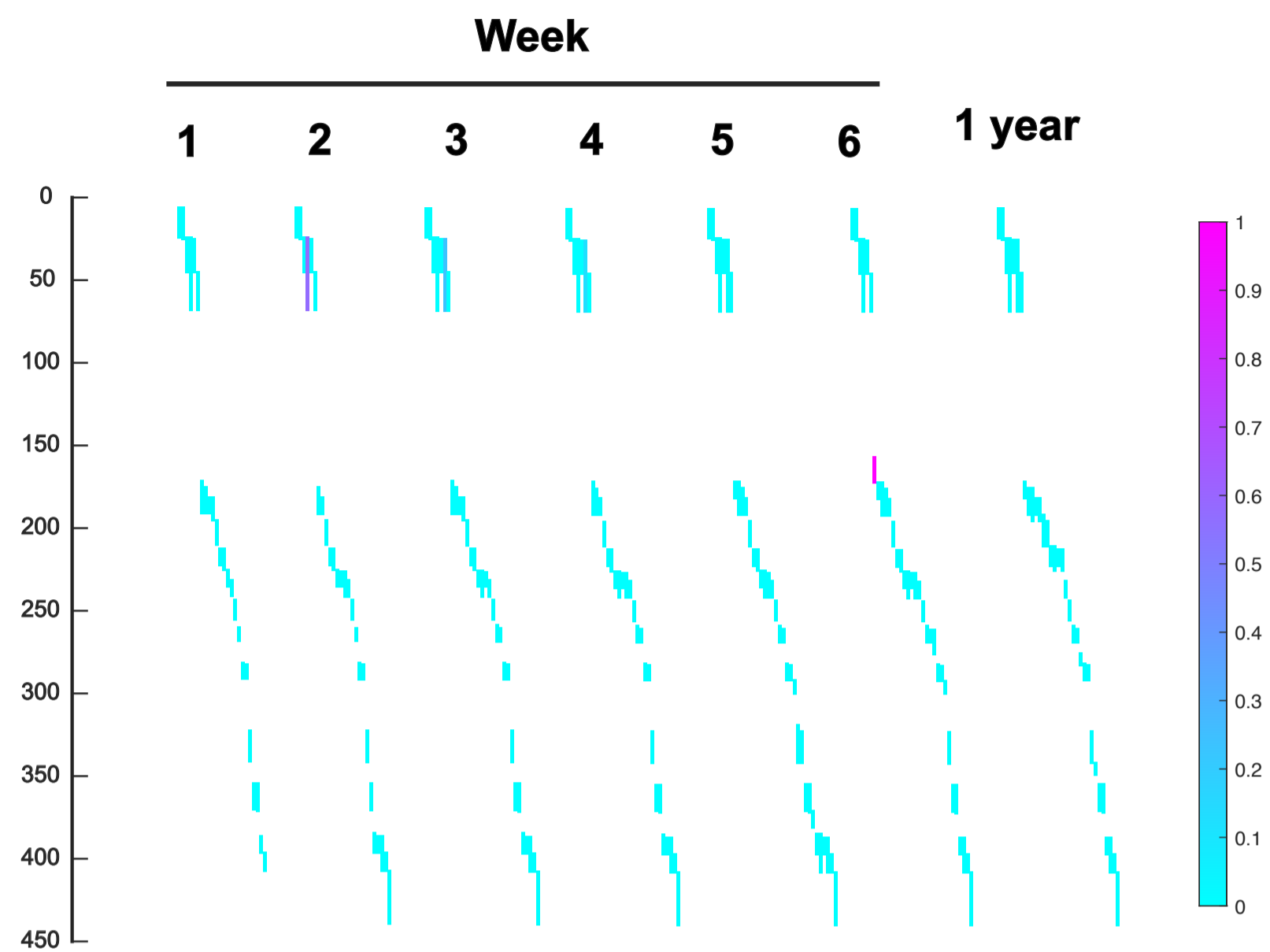**B**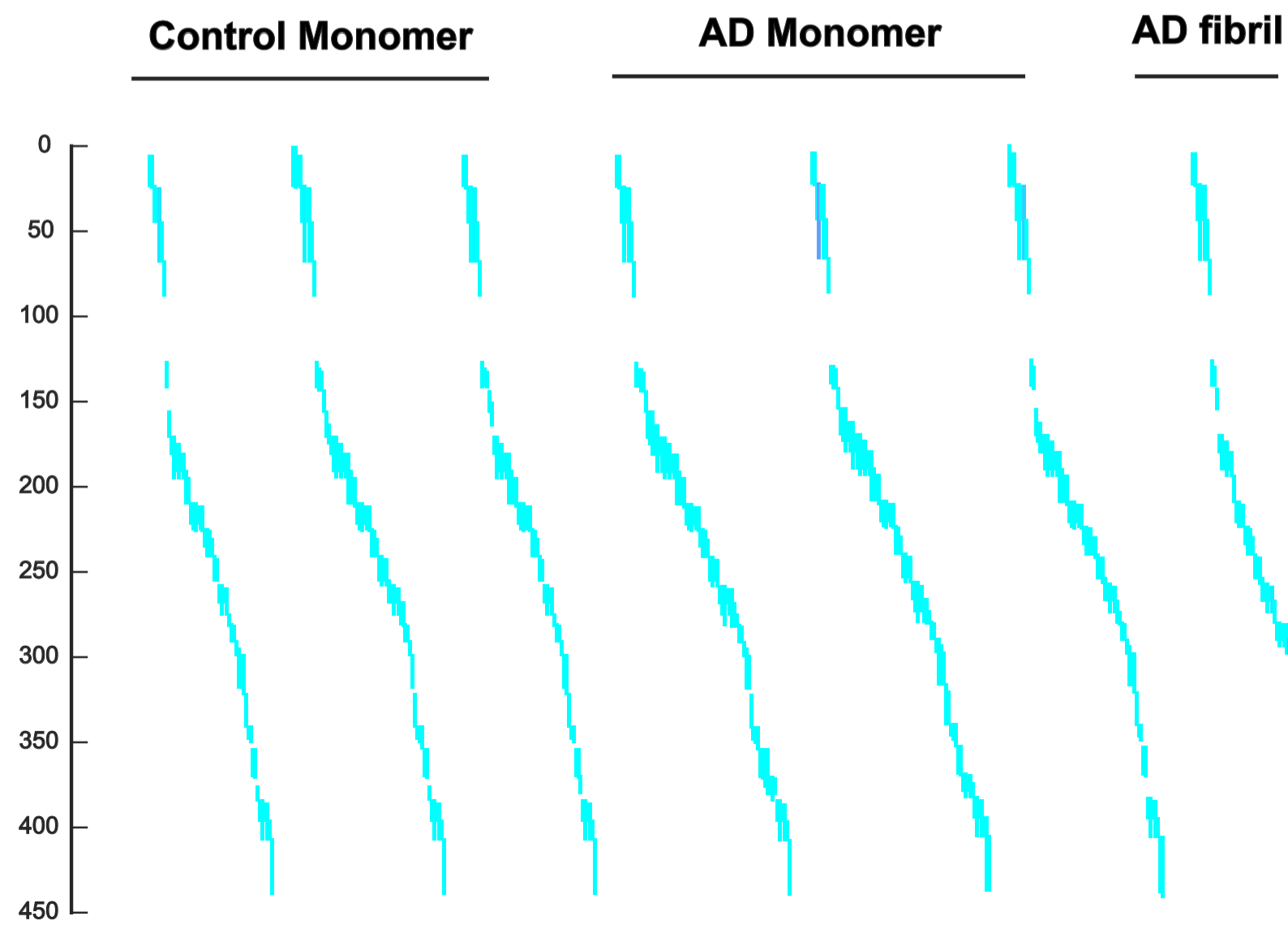**C**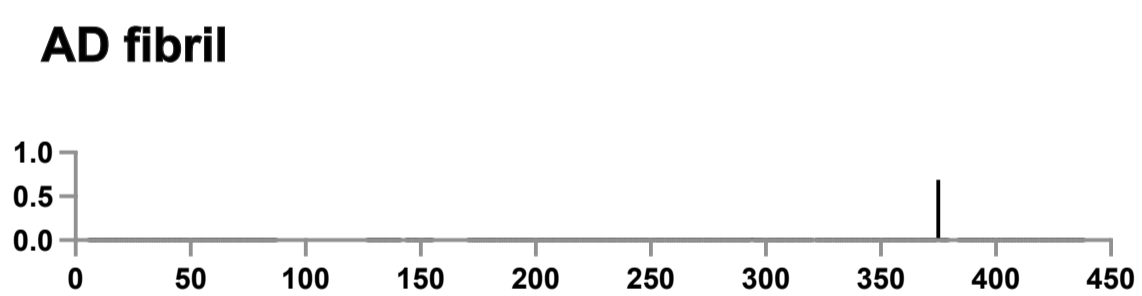

Supplement: Supplemental Figure S3-4 — Acetylation patterns for tau monomer isolated from PS19 mouse and human control and AD samples.A, peptide coverage map for tau monomer isolated from PS19 mice 1 to 6 weeks and 1 year. Peptide fragments are colored by acetylation frequency from 0 (cyan) to 100% (magenta). B, peptide coverage map for tau monomer isolated from age-matched controls, AD and AD fibrils. Peptide fragments are colored by acetylation frequency from 0 (cyan) to 100% (magenta). C, cumulative frequency of acetylation for specific sites across different peptide sequences for AD fibrils. Each bar represents a different peptide detected in the search, offset to allow discrimination. The search for tau peptides with and without acetylation post-translation modifications was carried out independently using the original mass spectrometry data. [file mmc6.pdf]

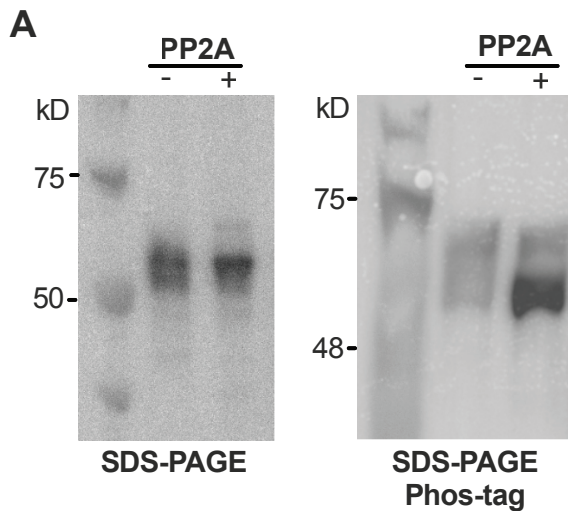

PS19 mouse brain

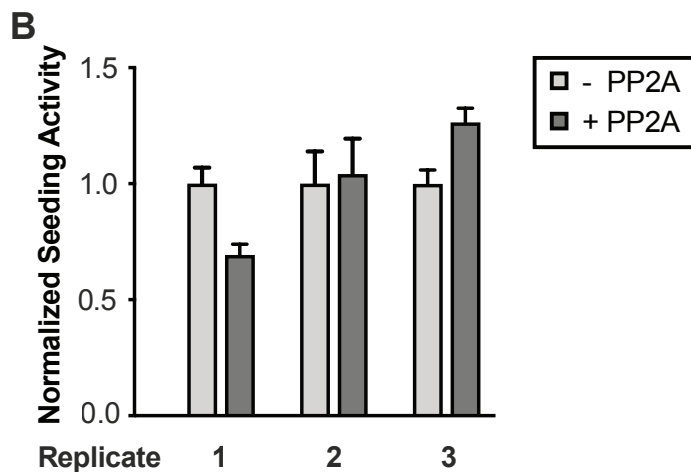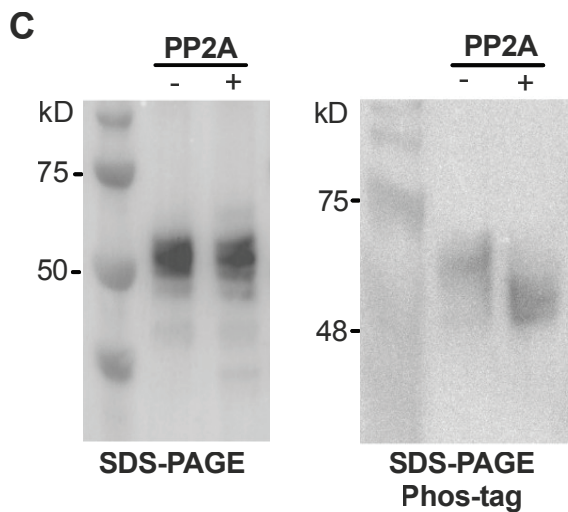

Alzheimer's Disease brain

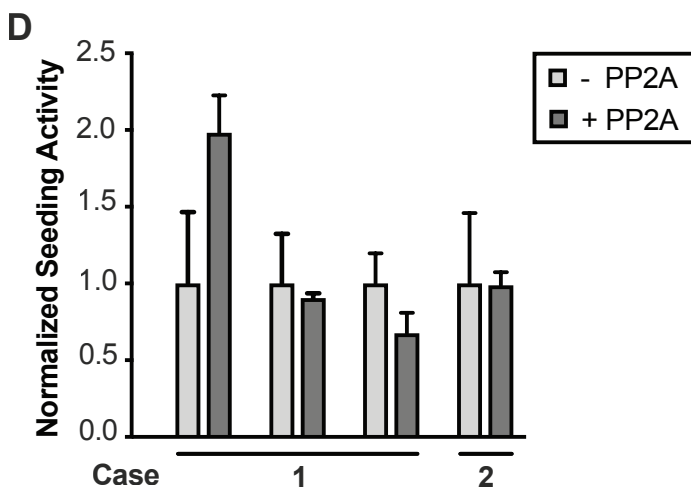

Supplement: Supplemental Figure S4 — Seeding activity of dephosphorylated tau monomer from AD and mouse.A, Western blot analysis of tau monomer isolated from PS19 mice resolved by SDS-PAGE and Phos-tag SDS-PAGE probed with anti-tau antibodies reveal patterns consistent with tau phosphorylation at multiple sites. Treatment of samples with phosphatase collapses bands on Phos-tag gel, indicating dephosphorylation. B, four independent replicates of tau monomer extracted from 1 year old PS19 mouse brains. Samples were treated with PP2A phosphatase (dark grey) and compared to non-treated control samples (grey) in a tau seeding assay. Error bars = S.D. C, Western blot analysis of tau monomer isolated from AD brains and resolved by SDS-PAGE and Phos-tag SDS-PAGE probed with anti-tau antibody. Treatment of samples with phosphatase collapses bands on Phos-tag gel, indicating dephosphorylation. D, four independent replicates of tau monomer were extracted from two different AD brains. Samples were treated with PP2A phosphatase (dark grey) and compared to non-treated samples (grey) in a tau seeding assay. Error bars = S.D. [file mmc7.pdf]
